# Posterior Reversible Encephalopathy Syndrome Presenting as Late Postpartum Eclampsia in a Previously Normotensive Woman: A Case Report

**DOI:** 10.7759/cureus.118000

**Published:** 2026-10-16

**Authors:** Kyaw Zin Aung, Naw Eh Law Saw

**Affiliations:** 1 Internal Medicine, Kulhudhuffushi Regional Hospital, Kulhudhuffushi, MDV

**Keywords:** generalized tonic-clonic seizures, hypertensive disorders of pregnancy (hdp), late postpartum eclampsia, posterior reversible encephalopathy syndrome (pres), reversible cerebral edema

## Abstract

Posterior reversible encephalopathy syndrome (PRES) is a clinico-radiological syndrome characterized by acute neurological manifestations, including headache and seizures, with neuroimaging evidence of predominantly reversible vasogenic cerebral edema. It is commonly associated with hypertensive disorders of pregnancy, particularly preeclampsia and eclampsia. A 27-year-old primigravida with known Hashimoto thyroiditis, treated with levothyroxine 50 µg daily, presented on postpartum day seven with a severe headache followed by two generalized tonic-clonic seizures. Her blood pressure was 160/110 mmHg, and the urine protein-to-creatinine ratio was 6.96 g/g creatinine. Magnetic resonance imaging (MRI) of the brain demonstrated bilateral parieto-occipital cortical and subcortical T2-weighted/fluid-attenuated inversion recovery (FLAIR) hyperintensities consistent with PRES. Magnetic resonance venography excluded cerebral venous sinus thrombosis. Autoimmune investigations for alternative systemic autoimmune disorders, including antinuclear antibody, anti-double-stranded DNA antibody, antineutrophil cytoplasmic antibody, and complement levels, were unremarkable. She was treated with magnesium sulfate and antihypertensive therapy, resulting in complete clinical recovery and resolution of the radiological abnormalities. This case highlights late postpartum eclampsia presenting as PRES in a previously normotensive woman and emphasizes the importance of maintaining a high index of suspicion for postpartum hypertensive disorders in women presenting with new-onset headache or seizures, even after an apparently uncomplicated pregnancy and delivery. Early recognition and prompt treatment are essential to prevent potentially life-threatening complications and achieve favorable clinical outcomes.

## Introduction

Posterior reversible encephalopathy syndrome (PRES) is a clinico-radiological syndrome characterized by acute neurological manifestations and distinctive neuroimaging findings of predominantly vasogenic edema, most commonly involving the parieto-occipital regions of the brain [1,2]. The pathophysiology is multifactorial and involves impaired cerebral autoregulation, endothelial dysfunction, and blood-brain barrier disruption [2].

PRES is associated with acute hypertension, renal dysfunction, sepsis, autoimmune disease, and exposure to immunosuppressive or cytotoxic therapies [2]. Among obstetric patients, preeclampsia and eclampsia are important precipitating factors. Eclampsia may occur antepartum, intrapartum, or postpartum and can develop in women without previously documented hypertension or preeclampsia [3,4].

Magnetic resonance imaging (MRI), particularly T2-weighted and fluid-attenuated inversion recovery (FLAIR) sequences, is the preferred imaging modality for evaluating suspected PRES. Typical findings include bilateral, often symmetrical vasogenic edema predominantly involving the parieto-occipital regions, although other areas of the brain may also be affected [2,5,6]. Early recognition and treatment are important because PRES is potentially reversible, with favorable clinical and radiological outcomes in most patients [1,7]. We report a case of PRES secondary to late postpartum eclampsia in a previously normotensive woman with Hashimoto thyroiditis who presented with severe headache and generalized tonic-clonic seizures seven days after an uncomplicated delivery.

## Case presentation

A 27-year-old primigravida presented on the seventh day after delivery with a one-day history of severe headache, followed by two episodes of generalized tonic-clonic seizures, each lasting approximately 1 min. The seizures were associated with tongue biting and urinary incontinence. Following the second episode, the patient experienced postictal confusion and regained consciousness approximately 30 min later. There was no history of fever, head trauma, visual disturbances, focal neurological symptoms, or aura. No persistent neurological deficits were identified after recovery.

The patient had an uneventful antenatal course and delivery, with no documented antenatal hypertension, preeclampsia, or associated warning symptoms in the available obstetric records. She underwent an elective lower-segment cesarean section (LSCS) at term at her request and was discharged in stable condition. Her medical history was significant for Hashimoto thyroiditis, diagnosed during the third trimester of pregnancy, for which she was receiving levothyroxine 50 µg daily. She was also taking iron and folic acid supplements.

On presentation, her blood pressure was 160/110 mmHg, with a heart rate of 88 beats/min and a respiratory rate of 16 breaths/min. Oxygen saturation was normal on room air. Neurological examination revealed no focal deficits, and her pupils were normal. There were no signs of meningeal irritation. Her blood glucose level was 143 mg/dL. Urinalysis demonstrated marked proteinuria, with an initial urine protein-to-creatinine ratio (PCR) of 6.96 g/g creatinine (reference range: <0.30 g/g creatinine).

Initial laboratory investigations revealed a capillary blood glucose level of 143 mg/dL and significant proteinuria, with a urine protein-to-creatinine ratio of 6.96 g/g creatinine, which gradually improved during hospitalization. Complete blood count, renal and liver function tests, serum electrolytes, coagulation profile, arterial blood gas analysis, and D-dimer showed no clinically significant abnormalities apart from mild anemia and an isolated elevation in alkaline phosphatase (Table 1). Autoimmune investigations, including antinuclear antibody (ANA), anti-double-stranded DNA antibody (anti-dsDNA), antineutrophil cytoplasmic antibody (ANCA), and complement components C3 and C4, did not suggest an alternative systemic autoimmune disorder (Table 2). Anti-thyroid peroxidase antibody levels were elevated, consistent with her known Hashimoto thyroiditis.

**Table 1:** Laboratory investigations on admission. FEU: fibrinogen equivalent units

Investigation	Result on admission	Reference range
Hemoglobin (Hb)	11.0 g/dL	12.0-16.0 g/dL
White blood cell count (WBC)	7.2×10^9^/L	4.0-11.0×10^9^/L
Platelet count	174×10^9^/L	150-400×10^9^/L
Erythrocyte sedimentation rate (ESR)	17 mm/h	0-20 mm/h
C-reactive protein (CRP)	8.1 mg/L	<10 mg/L
Sodium (Na⁺)	138 mmol/L	137-145 mmol/L
Potassium (K⁺)	4.4 mmol/L	3.5-5.1 mmol/L
Serum calcium	8.8 mg/dL	8.2-10.2 mg/dL
Serum magnesium	2.2 mg/dL	1.6-2.3 mg/dL
D-dimer	0.42 mg/L FEU	<0.50 mg/L FEU
Urine protein-to-creatinine ratio	6.96 g/g creatinine	<0.30 g/g creatinine
Total bilirubin	0.6 mg/dL	0.2-1.3 mg/dL
Aspartate aminotransferase (AST)	34 U/L	14-36 U/L
Alanine aminotransferase (ALT)	26 U/L	<35 U/L
Alkaline phosphatase (ALP)	211 U/L	38-126 U/L
Blood urea	25.0 mg/dL	15.0-40.0 mg/dL
Uric acid	3.0 mg/dL	2.5-6.2 mg/dL
Creatinine	0.50 mg/dL	0.52-1.04 mg/dL
Triiodothyronine (T3)	1.83 nmol/L	1.1-3.1 nmol/L
Thyroxine (T4)	132.0 nmol/L	55-160 nmol/L
Thyroid-stimulating hormone (TSH)	2.28 mIU/L	0.47-4.94 mIU/L

**Table 2:** Autoimmune investigation results.

Investigation	Result on admission	Reference value
Antinuclear antibody (ANA)	0.60 index - negative	<1.0 index
Anti-double-stranded DNA antibody (anti-dsDNA)	<1.0 index - negative	<1.0 index
Complement component 3 (C3)	134 mg/dL	83-193 mg/dL
Complement component 4 (C4)	29.1 mg/dL	15-57 mg/dL
Anti-thyroid peroxidase antibody (anti-TPO antibody)	13.7 IU/mL	0.0-5.60 IU/mL
Antineutrophil cytoplasmic antibody (ANCA)	Negative	Negative

Brain MRI demonstrated bilateral, relatively symmetrical cortical and subcortical hyperintensities involving the medial parietal and occipital lobes on T2-weighted and FLAIR sequences, consistent with PRES (Figures 1A, 1B). Magnetic resonance venography showed no evidence of cerebral venous sinus thrombosis. Electroencephalography (EEG) showed a posterior dominant alpha rhythm of 9-10 Hz with generalized theta slowing predominantly in the posterior parietal regions, suggestive of mild posterior cortical dysfunction (Figure 2). Hyperventilation and photic stimulation were not performed, and sleep recording was not obtained.

**Figure 1:**
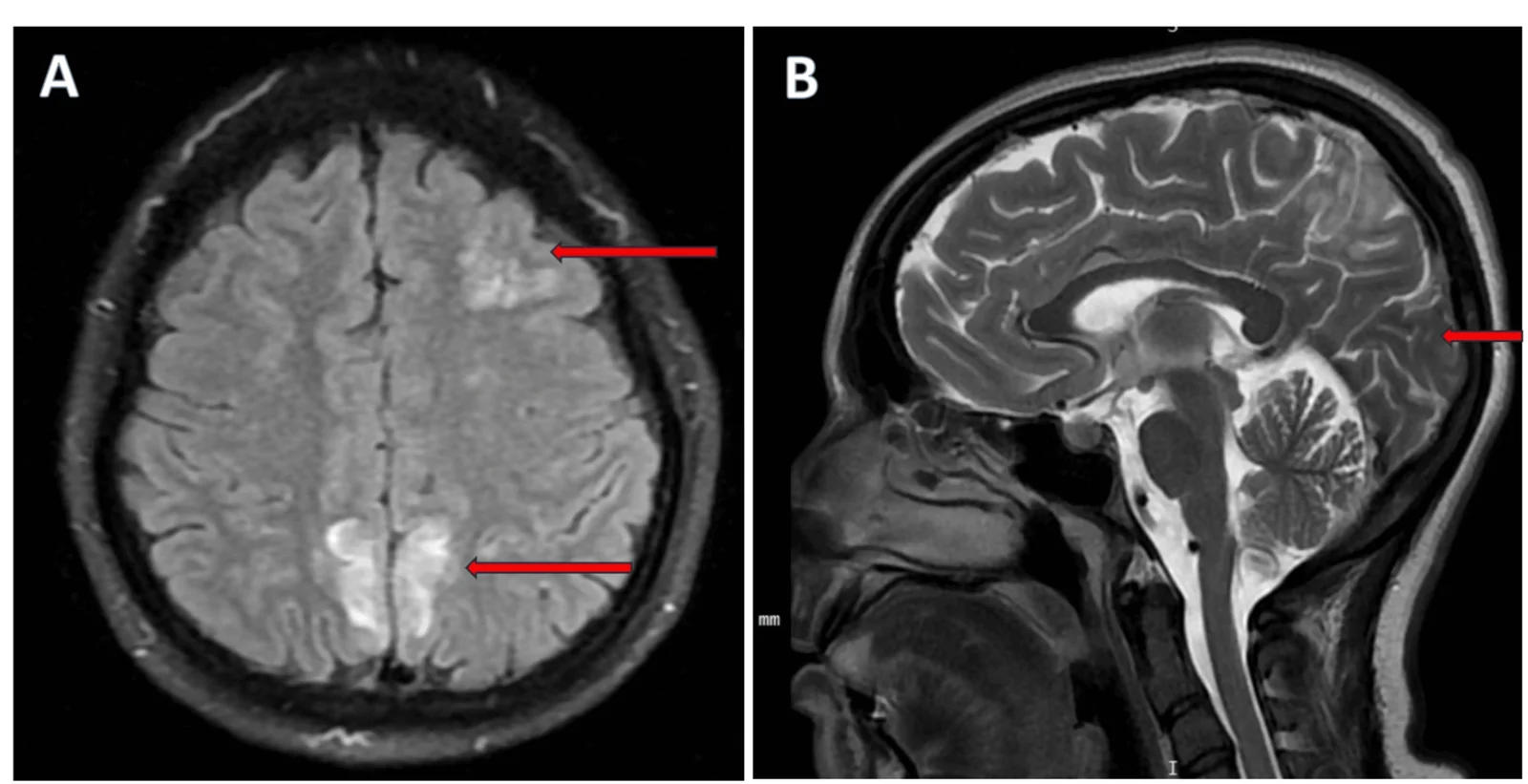
Magnetic resonance imaging findings in posterior reversible encephalopathy syndrome (PRES). (A) Axial FLAIR image demonstrating bilateral parieto-occipital cortical and subcortical hyperintensities (arrows), consistent with vasogenic edema. (B) Sagittal T2-weighted image demonstrating posterior-predominant hyperintense signal involving the parieto-occipital regions (arrow). FLAIR: fluid-attenuated inversion recovery

**Figure 2:**
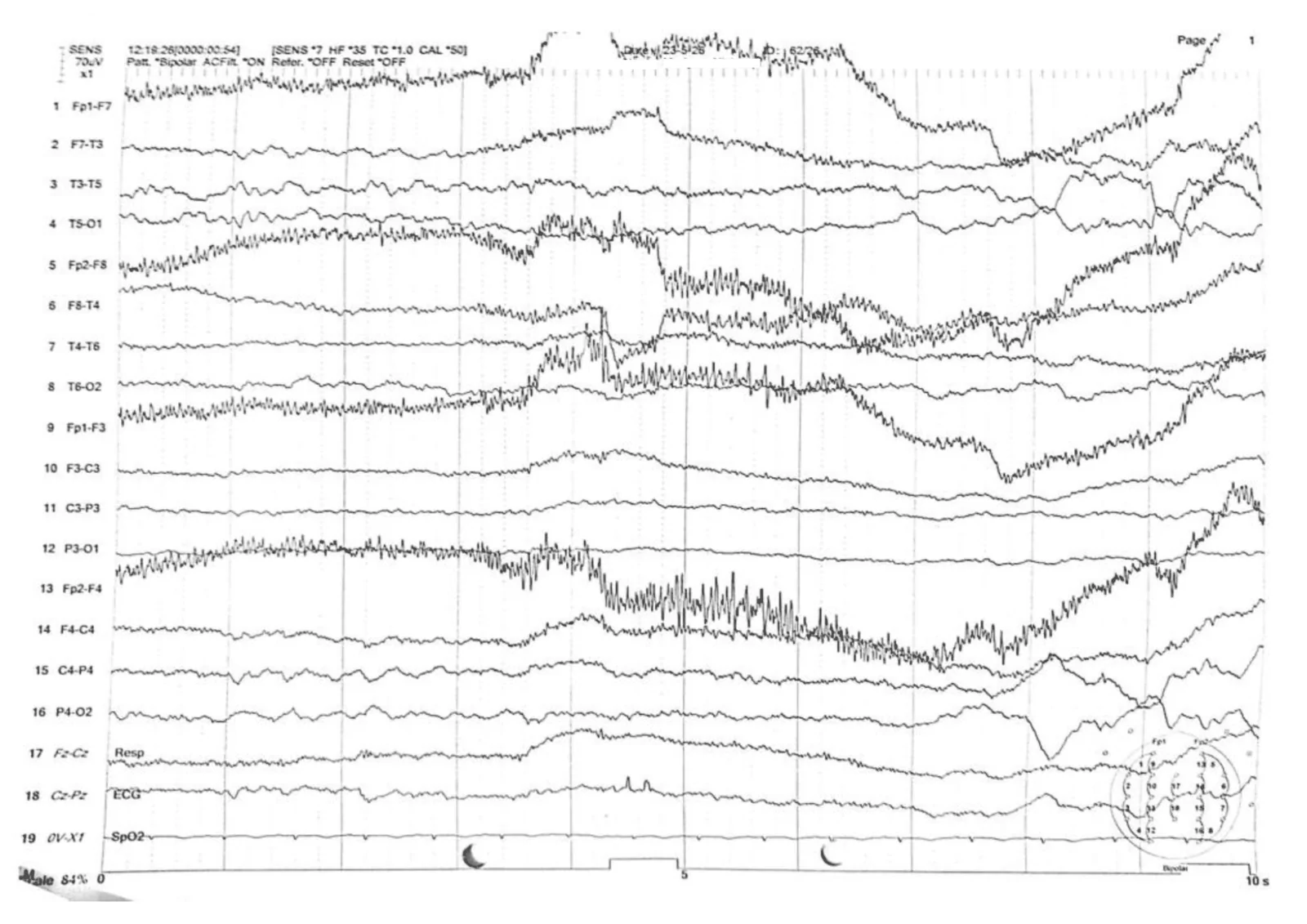
Electroencephalography showing posterior-predominant generalized theta slowing. Electroencephalography (EEG) demonstrates a posterior dominant alpha rhythm of approximately 9-10 Hz with generalized theta slowing, more prominent over the posterior regions, suggestive of mild diffuse cerebral dysfunction with posterior predominance. SENS: sensitivity; HF: high-frequency filter; TC: time constant; CAL: calibration

Based on the clinical presentation, severe hypertension, significant proteinuria, and characteristic neuroimaging findings, a diagnosis of posterior reversible encephalopathy syndrome secondary to late postpartum eclampsia was made. The patient was treated with intravenous labetalol for acute blood pressure control and magnesium sulfate for seizure management and prophylaxis. Magnesium sulfate was administered as a 4-g intravenous loading dose, followed by a continuous infusion at 1 g/h for 24 h. Oral nifedipine was subsequently initiated, and the patient was admitted to the intensive care unit for close monitoring. No further seizures occurred, and her blood pressure gradually normalized. Serial measurements demonstrated sustained improvement in blood pressure and a progressive reduction in urine protein-to-creatinine ratio (PCR) during hospitalization and follow-up (Table 3).

**Table 3:** Serial blood pressure and urine protein-to-creatinine ratio measurements. PCR: urine protein-to-creatinine ratio

Time point	Blood pressure (mmHg)	Urine PCR (g/g creatinine)
Presentation (day 0)	160/110	6.96
Day 1	140/80	5.2
Day 2	120/72	4.9
Day 3	110/70	3.6
Day 5	110/60	2.4
Day 7 (1 week)	110/60	0.3
Day 14 (2 weeks)	110/70	0.2

The patient remained neurologically intact throughout her admission and was discharged on the fifth hospital day with nifedipine 10 mg twice daily. At the one-week follow-up, her blood pressure normalized, antihypertensive therapy was discontinued, and repeat urinalysis showed complete resolution of proteinuria. Follow-up MRI performed two weeks later showed complete resolution of the previously identified lesions, further supporting the diagnosis of PRES.

## Discussion

Posterior reversible encephalopathy syndrome (PRES) is a potentially reversible neurological condition characterized by acute neurological symptoms and vasogenic cerebral edema, most commonly associated with hypertension and endothelial dysfunction [1,2]. Its pathophysiology is thought to involve impaired cerebrovascular autoregulation and disruption of the blood-brain barrier, resulting in vasogenic edema. The posterior circulation may be particularly vulnerable because of its relatively limited sympathetic innervation, although the underlying mechanisms are multifactorial [2,3].

Eclampsia is characterized by new-onset seizures associated with a hypertensive disorder of pregnancy when alternative causes are not identified. It may occur antepartum, intrapartum, or postpartum, including in women without previously documented hypertension or preeclampsia [4]. Late postpartum eclampsia is particularly challenging to recognize because symptoms may develop after hospital discharge. In the present case, the patient had an apparently uncomplicated pregnancy without documented antenatal hypertension or preeclampsia but presented on postpartum day seven with severe headache, generalized tonic-clonic seizures, severe hypertension, and marked proteinuria. This presentation highlights the importance of considering postpartum eclampsia even when the antenatal course has been uneventful.

MRI is the preferred imaging modality for suspected PRES, typically demonstrating bilateral parieto-occipital hyperintensities on T2-weighted and fluid-attenuated inversion recovery (FLAIR) sequences, consistent with vasogenic edema [2,5,6]. Although the parieto-occipital regions are most frequently affected, other areas, including the frontal lobes, cerebral cortex, brainstem, and cerebellum, may also be involved [6]. In our patient, MRI demonstrated bilateral, relatively symmetrical abnormalities involving the medial parietal and occipital regions. Complete resolution of these abnormalities on follow-up MRI two weeks later further supported the diagnosis [7].

The differential diagnosis of postpartum seizures includes cerebral venous sinus thrombosis, intracranial hemorrhage, metabolic disturbances, epilepsy, and infectious or autoimmune encephalitis. In this case, magnetic resonance venography excluded cerebral venous sinus thrombosis, while the clinical assessment, laboratory investigations, and negative autoimmune screening did not support an alternative systemic autoimmune cause. The characteristic MRI findings, severe postpartum hypertension, marked proteinuria, and subsequent radiological resolution collectively supported PRES associated with late postpartum eclampsia. The EEG demonstrated posterior-predominant slowing, consistent with nonspecific cerebral dysfunction. Although EEG abnormalities may occur in PRES, they are not diagnostic and must be interpreted alongside clinical and neuroimaging findings [7].

Management of eclampsia-associated PRES requires prompt seizure management, controlled reduction of severe hypertension, and supportive care [2,8]. Magnesium sulfate is established as the preferred treatment for eclamptic seizures and is effective in reducing the risk of eclampsia in women with preeclampsia. The Magpie Trial demonstrated a substantial reduction in the occurrence of eclampsia with magnesium sulfate prophylaxis [9]. Evidence also supports magnesium sulfate over diazepam for the treatment of eclamptic convulsions [8]. In the present case, magnesium sulfate was administered as a 4-g intravenous loading dose followed by an infusion of 1 g/h for 24 h. Intravenous labetalol was used for acute blood pressure control, followed by oral nifedipine. The patient experienced no further seizures, and her blood pressure progressively normalized.

PRES generally has a favorable prognosis when the underlying cause is recognized and treated promptly, although delayed treatment or severe complications may result in permanent neurological injury [1,7]. In this case, the patient achieved complete clinical recovery without persistent neurological deficits, accompanied by complete resolution of the MRI abnormalities within two weeks. Serial measurements also demonstrated progressive improvement in blood pressure and a substantial decline in urine protein-to-creatinine ratio during follow-up. These findings were consistent with a favorable response to treatment.

Hashimoto thyroiditis was an additional clinical feature in this patient. However, the available findings do not establish a causal relationship between the thyroid disorder and the development of postpartum eclampsia or PRES. Therefore, its presence should be interpreted as a comorbidity rather than a demonstrated contributing mechanism.

This case highlights the importance of maintaining a high index of suspicion for late postpartum eclampsia and PRES in women presenting with severe headache and seizures after delivery, even in the absence of documented antenatal hypertension or preeclampsia. Prompt clinical assessment, appropriate neuroimaging, magnesium sulfate therapy, and effective blood pressure control are essential for early diagnosis, prevention of further seizures, and favorable neurological outcomes.

## Conclusions

This case illustrates posterior reversible encephalopathy syndrome secondary to late postpartum eclampsia in a previously normotensive woman with Hashimoto thyroiditis. It highlights the importance of considering eclampsia and PRES in postpartum patients presenting with severe headache and seizures, even in the absence of antenatal hypertension or preeclampsia. Prompt recognition, appropriate neuroimaging, magnesium sulfate therapy, and blood pressure control are essential to prevent neurological complications and achieve favorable outcomes.
